# Incidence and Mortality Risks of Cancer in Patients with Type 2 Diabetes: A Retrospective Study in Shanghai, China

**DOI:** 10.3390/ijerph13060559

**Published:** 2016-06-03

**Authors:** Yunjuan Gu, Xuhong Hou, Ying Zheng, Chunfang Wang, Lei Zhang, Jie Li, Zhezhou Huang, Ming Han, Yuqian Bao, Weijian Zhong, Weiping Jia, Shiwei Cui

**Affiliations:** 1 Department of Endocrinology and Metabolism, Affiliated Hospital of Nantong University, Nantong 226001, China; desette010@aliyun.com; 2 Shanghai Key Laboratory of Diabetes Mellitus, Shanghai 200233, China; houxuhongsjtu@163.com (X.H.); guojieli73@163.com (J.L.); byq522@163.com (Y.B.); 3 Shanghai Diabetes Institute, Shanghai 200233, China; 4 Shanghai Clinical Centre of Diabetes, Shanghai 200233, China; stonez78@hotmail.com; 5 Department of Cancer Prevention and Control, Shanghai Municipal Center for Disease Control and Prevention, Shanghai 200233, China; zhengying@scdc.sh.cn (Y.Z.); huangzhezhou@scdc.sh.cn (Z.H.); 6 Department of Vital Statistics, Shanghai Municipal Center for Disease Control and Prevention, Shanghai 200233, China; wangchunfang@scdc.sh.cn (C.W.); hanming@scdc.sh.cn (M.H.); 7 Department of Endocrinology and Metabolism, Shanghai Jiao Tong University Affiliated Sixth People’s Hospital, Shanghai 200233, China; 8 Vice-Director of the Shanghai Municipal Center for Disease Control and Prevention, Shanghai 200233, China; zhongweijian@scdc.sh.cn

**Keywords:** type 2 diabetes, cancer, mortality, standardized incidence ratio, standardized mortality ratio

## Abstract

*Background:* Evidence from epidemiologic investigation indicates that people with type 2 diabetes (T2DM) are at a significantly higher risk of many types of cancer and mortality. The aim of this study was to investigate the incidence and mortality risks of cancer in patients with T2DM compared with the general population in Shanghai, China. *Methods*: Based on the Shanghai Diabetes Registry (SDR) database linking to the Shanghai Cancer Registry and Surveillance System (SCRSS), a total of 12,276 T2DM patients without cancer were defined and followed up from 1 December 2001 to 31 July 2011. Standardized incidence ratio (SIR) and standardized mortality ratio (SMR) with 95% confidence interval (CI) were calculated using the whole gender and age-matched general population of Shanghai as a reference during the same period. *Results*: The overall cancer risk was found higher in both males and females T2DM patients, with the SIR of 3.14 (95% CI 2.73–3.56) and 4.29 (95% CI 3.64–4.94), respectively. The overall mortality risk of cancer also significantly increased with the SMR of 2.27 (95% CI 1.86–2.68) and 1.86 (95% CI 1.46–2.26), respectively. Pancreatic cancer was with the highest SIR and SMR in both genders. *Conclusions*: Compared with the general population, patients with T2DM were associated with higher incidence and mortality risks of cancer, especially pancreatic cancer.

## 1. Introduction

Both diabetes and cancer are prevalent diseases with globally increasing incidence [1]. They are two chronic heterogeneous diseases, although they share many common risk factors including age, sex, obesity, physical activity, diet, alcohol and smoking [1]. Evidence from epidemiological studies and meta-analysis has clearly indicated that the risk of cancer was significantly increased in diabetic patients, predominantly in T2DM [2,3,4]. Recently, several cohort studies in Chinese patients demonstrated that T2DM was associated with an increased risk of colorectal [5] and liver cancer [6]. In addition, a Chinese population-based retrospective cohort study carried out in Zhejiang Province showed an increased risk of overall cancer in T2DM patients, with a SIR of 1.331 (95% CI = 1.143–1.518) for men and 1.737 (95% CI = 1.478–1.997) for women, respectively [7]. Furthermore, the existence of diabetes mellitus confers risk of substantial premature death from some cancers [8]. Recent analysis on individual participant data from 97 prospective studies found that diabetic patients had a higher mortality risk of liver, pancreatic, ovarian, colorectal, lung and breast cancers [8].

To date, knowledge about the difference in cancer and mortality risks between T2DM patients and the general population in Mainland China have not been evaluated simultaneously. In the present study, we aimed to investigate the association between T2DM and incidence and mortality of cancer compared with the whole general population-based risk in Shanghai, China.

## 2. Methods

### 2.1. Study Population

This was a population-based, retrospective study performed by using the Shanghai Diabetes Registry (SDR) database, which was established in December 2001 at the Shanghai Jiao Tong University Affiliated Sixth People’s Hospital, the Shanghai Clinical Center for Diabetes. This computerized database was set up with the aim of evaluating the outcomes of Chinese patients with diabetes. The participants in our study included outpatients and hospitalized patients were all residents in Shanghai. With their consents to participate in this follow-up program, they completed a registry questionnaire and received a comprehensive assessment of diabetic complications. After being assigned to a unique registry number, each rerolled patient was invited to attend follow-up visit at the center throughout their lifetime. Demographics, diagnoses, laboratory measurements, and other detailed information of each subject were recorded by nurses trained in the care of patients with diabetes [9,10]. From 1 December 2001 to 31 July 2010, there were 12,973 Shanghai residents (>20 years old) with diabetes were enrolled in the database. After excluding 181 patients with type 1 diabetes, gestational diabetes, secondary diabetes and 516 patients who had been diagnosed with cancer at baseline, a total of 12,276 T2DM patients were selected and followed up until 31 July 2011.

In Shanghai, all general hospitals and community health service centers are obligated to register cancer events and mortalities to the Shanghai Municipal Centre for Disease Control and Prevention (SHCDC). The data was then recorded in the Shanghai Cancer Registry and Surveillance System (SCRSS) database, which was established in January 2002 and covers all regions of Shanghai. The rate of leakage is less than 5% in the SCRSS database.

Based on the unique identification number, we extracted the patients with T2DM who developed cancer and/or died by linking SDR and SCRSS database during the follow-up time.

Identification of cancer types, sites, and death was performed according to the International Classification of Diseases, 10th revisions (ICD-10). Common cancers in this study, identified by ICD-10 codes, included lung (C33–C34), colorectal (C18–C20), gastric (C16), liver (C22), pancreatic (C25), breast (C50) and prostate (C61).

### 2.2. Statistical Analyses

Person-years of follow-up of each T2DM patient were calculated from the date the T2DM patient was registered to the date when site-specific cancer was diagnosed, or when death occurred or when the study was completed (31 July 2011), whichever occurred first. Chi-square test was used to compare categorical variables between male and female patients. The Cox regression model was performed to estimate the hazard ratios of cancer and mortality between genders by adjusting age, gender, smoking, diabetes duration, HbA1c, and systolic blood pressure (SBP). The incidence rates of overall and specific cancer and mortality were calculated by the number of incidence cases divided by the number of observed person-years. The standardized incidence ratio (SIR) and standardized mortality ratio (SMR) with their 95% confident intervals (CI) were calculated as the ratio of observed to expected cases number. The expected numbers of cancer or mortality occurrences were calculated by multiplying the cancer incidence or mortality rate in the general population (retrieved from Shanghai Cancer Registry and Surveillance System database) according to gender, calendar year and age in 5-year intervals by the corresponding stratum-specific person-time accrued in the cohort [11]. The 95% confidence intervals (CI) for SIR and SMR were then calculated using the following formula: 1.96 ± 1.96 Se, where Se = SIR/$\sqrt{\text{number of observed cases}}$ or Se = SMR/$\sqrt{\text{number of observed cases}}$ [7]. All analyses were performed using Excel 2007 (Microsoft Corporation, Seattle, WA, USA) and SPSS statistical package version 16.0 (SPSS, Chicago, IL, USA).

### 2.3. Ethics Statement

This study was approved by the Ethics Committee of Shanghai Jiao Tong University Affiliated Sixth People’s Hospital in accordance with the Declaration of Helsinki (No. 2015-26). All subjects gave their informed consent for inclusion before they participated in the study.

## 3. Results

### 3.1. Basic Characteristics and Follow-Up Time

Characteristics of the T2DM patients at baseline are shown in Table 1. Among the 12,276 T2DM patients, 6488 (52.9%) were male and 5788 (47.1%) were female. The female patients were significantly older than the males (median (inter quartile range, IQR): 61.9 (17.0) *vs.* 60.2 (18.0), *p* < 0.0001). The ratio of smoking patients was significantly higher in male patients than that of female ones (40.3% *vs.* 2.0%, *p* < 0.0001). More females had diabetic family history than that of males (39.2% *vs.* 37.0%, *p* = 0.008). Compared with males, the level of SBP was significantly higher and HbA1c was significantly lower in females (all *p* < 0.0001). No significant difference was found in the level of BMI between male and female patients. The percentages of micro- and macro-vascular complications were similar in the two genders.

Table 2 describes the follow-up time of the T2DM patients. The median (mean) follow-up time was 4.2 (4.7) years in male patients and 4.2 (4.6) years in female patients, respectively. The highest ratio of patients was in the age range of 55–64 years (29.8%), with the follow-up time of 16,062.3 person years. At baseline, the median diabetic durations was 5.4 years with the IQR of 9.4 years. The follow-up time of the patients with the diabetic duration ≥10 years was longest, which was 16,391.2 person years.

### 3.2. Incidence Rates of Cancer and Mortality in Male and Female Patients

As shown in Table 3, 404 cancer events (226 in males and 178 in females) were observed, with an overall cancer incidence rate of 774.1 and 677.8 per 10^5^ person per year in male and female T2DM patients, respectively. For site-specific cancer, the highest incidence rate was found in colorectal cancer (116.5 per 10^5^ person per year) in males and lung cancer (121.9 per 10^5^ person per year) in females, respectively. Compared with male patients, females had significantly lower risks of colorectal cancer (*p* = 0.031) and liver cancer (*p* = 0.005), with crude HRs of 0.52 (95% CI 0.29–0.94) and 0.36 (95% CI 0.17–0.75), respectively. However, after adjusting for age, smoking, diabetes duration, HbA1c and SBP, risks of overall or site-specific cancers were not significantly different between male and female T2DM patients.

Within the study, 411 male and 368 female T2DM patients died. Among them, 113 males and 83 females died of cancer, with a mortality rate of 386.2 per 10^5^ patients per year and 315.6 per 10^5^ person per year, respectively. For site-specific cancer, lung cancer was with the highest mortality rate in both male (82.0 per 10^5^ patients per year) and female (87.5 per 10^5^ person per year) patients. No significant differences were found in the mortality risks of all-causes, overall cancer or site-specific cancer between male and female patients, both in crude and adjusted models.

### 3.3. Standard Incidence Ratio of Cancer

As shown in Table 4, compared with the general population, T2DM patients had significantly higher overall cancer risk, with the SIR of 3.14 (95% CI: 2.73–3.56) in males and 4.29 (95% CI, 3.64–4.94) in females, respectively. For site–specific cancer, pancreatic cancer exhibited the highest SIR of 5.46 (95% CI, 2.69–8.22) in males and 9.00 (95% CI, 4.59–13.41) in females, respectively. Risks of lung, colorectal, gastric and liver cancer all significantly increased in T2DM patients, with the SIRs of 1.74 (95% CI, 1.13–2.34), 3.93 (95% CI, 2.61–5.24), 3.13 (95% CI, 2.06–4.21) and 3.86 (95% CI, 2.43–5.29) in males, respectively, and 5.71 (95% CI, 3.74–7.69), 2.56 (95% CI, 1.30–3.81), 4.19 (95% CI, 2.25–6.13) and 3.56 (95% CI, 1.15–5.97) in females, respectively. For different gender, the risk of prostate and breast cancer increased in males and females, with the SIR of 5.38 (95% CI, 3.01–7.05) and 4.60 (95% CI, 2.90–6.31), respectively.

### 3.4. Standard Incidence Ratio of Mortality

The SMRs of all–cause and cancer related mortality were shown in Table 5. Increased risk of all–cause mortality was found in both male and female T2DM patients, with the SMR of 1.88 (95% CI, 1.70–2.07) and 3.57 (95% CI, 2.78–4.35), respectively. The risk of overall cancer mortality significantly increased, with the SMR of 2.27 (95% CI, 1.86–2.68) in males and 1.86 (95% CI, 1.46–2.26) in females, respectively. For site–specific cancer, pancreatic cancer had the highest SMR of 4.35 (95% CI, 1.88–6.82) in male and 7.76 (95% CI, 3.83–11.69) in female patients, respectively. Additionally, mortality risks related to gastric cancer (SMR=2.14, 95% CI, 1.06–3.22) and liver cancer (SMR = 3.28, 95% CI, 1.85–4.71) significantly increased in male patients, while lung cancer (SMR = 4.90, 95% CI, 2.90–6.90) significantly increased in female patients.

## 4. Discussion

In the present study, we found that compared with the general population, T2DM patients had significantly higher incidence and mortality risks of cancer. Pancreatic cancer was with the highest SIR and SMR in both genders. The risks of overall and site-specific cancer and mortality were not significantly different between male and female T2DM patients.

Several hypotheses have been proposed to explain the complicated mechanism of higher incidence and mortality risks of cancer in T2DM patients. Firstly, evidence from observational studies suggested that poor controlled hyperglycemia has a potential effect on tumorigenesis and mortality. Yang *et al.* found that HbA1c was positively associated with an increased cancer risk (HR = 1.26, 95% CI 1.03–1.55) [12]. A systematic review evaluated 19 studies and reported that HbA1c was associated with cancer incidence and/or cancer mortality [13]. Another research focused on individual participant data from 97 prospective studies indicated that there are generally associations between fasting glucose levels greater than 100 mg per deciliter and risk of cancer death [8]. Secondly, hyperinsulinemia exists in most T2DM patients during the early period of diabetes, and might be another potential cancer risk due to its role in biological cell proliferation via the IGF–1 signaling pathway [14]. Thirdly, evidence from observational studies suggested that some hypoglycemia agents might have relationships with either increased or reduced cancer risk. Results of a growing number of studies suggested that treatment with metformin was associated with reduced risk and mortality of cancer [15,16]. However, the associations between cancer, mortality risks and other antidiabetic drugs, such as insulin [9,12,17,18], sulfonylureas [19,20] and thiazolidinedione [21,22], were controversial. Finally, many other confounders including diabetic duration, inflammation status, smoking, obesity, toxins and virus might contribute to oncogenesis and mortality in T2DM [1,23,24].

In China, several population based studies have reported that the SIR was higher in females than in males [7,25,26]. In our study, the higher SIR in females was also observed. The reason might be the contribution of the higher SIRs of lung cancer, gastric cancer and pancreatic cancer in females, which could be confirmed by a recent prospective research in Chinese T2DM patients [26]. However, the overall SMR was higher in males than that of females. It might due to the higher mortality in liver, pancreatic and colorectal cancer, which listed in the top four ranked cancer according to mortality [8].

A large number of studies reported that compared with the general or non–diabetic populations, patients with T2DM reportedly had higher risks of several common cancers including lung cancer [27], liver cancer [6], pancreatic cancer [28], colorectal cancer [29], gastric cancer [30] and breast cancer [31]. In our study, we found that the risks of these common cancers were all significantly increased too.

However, the relationship between diabetes and prostate cancer remains uncertain [32]. Heterogeneity and complicated scenario may be the reasons for the divergent relations between diabetes and prostate cancer in different ethnic group. Although studies and meta–analyses basing on western population data found an inversed relationship between prostate cancer and male T2DM patients [33,34], Tseng declared a positive relationship between prostate cancer and T2DM in Chinese males [35]. Ohsaki Cohort Study in Japan observed that a history of diabetes was not associated with the risk of total prostate cancer, however diabetic patients had a higher risk of advanced prostate cancer [36]. Leitzmann *et al.* found that diabetes was inversely associated with early stage prostate cancer, but it showed a positive association between diabetes and aggressive prostate cancer in the subgroup of men with a low BMI [37].The relation between diabetes and different stages of prostate cancer was absent because the information of prostate cancer stages in the database used in our study was insufficient for analysis. Additionally, the prostate antigen test has been used for prostate cancer screening among old men in a large number of hospitals in Shanghai, which may be a possible screening bias in our study.

In the present study, both male and female T2DM patients had the highest incidence and mortality risks of pancreatic cancer. It should be cautious that there was a bidirectional relationship between diabetes and pancreatic cancer. Multiple epidemiological studies and meta–analysis have showed that type 2 diabetes especially new–onset diabetes was associated with an increasing risk of pancreatic cancer [1,38,39,40] and pancreatic cancer could cause diabetes because of islet destruction. Although the patients who had any pancreatic diseases before the diagnosis of T2DM were excluded in our study, the possible bias still might not be eliminated at all.

The possible mechanisms for the strong association between diabetes and pancreatic oncogenesis remain unclear. Additionally, pancreatic cancer is now the fourth leading cause of cancer mortality in the USA [41], and will be the leading cause by 2050 [42]. More than 95% of all patients succumb to the disease within two years since diagnosis [43] due to advanced stage at diagnosis and ineffective therapy [44], so it is important to detect the disease as early as possible. Carbohydrate antigen 19-9 (CA 19-9) is the only pancreatic cancer biomarker which is widely used for approximately 30 years. It is suggested that early check–up of CA 19-9 could be useful for pancreatic cancer screening in asymptomatic patients with new–onset diabetes in the first 2 years [45]. However, there are several limitations in detecting pancreatic cancer by using CA 19-9, such as some small pancreatic cancer is with normal level [46], some T2DM patient without pancreatic cancer has positive result [47]. Therefore, more potential accurate biomarkers are expected to explore in future.

There are some limitations in our study. Firstly, the data of T2DM was only collected from one center in Shanghai, one of the most developed regions in China. Therefore, the results could not represent all the patients in Shanghai. Secondly, it had been reported that there were several potential risk factors common to both cancer and diabetes including age, gender, obesity, physical activity, diet, alcohol, and smoking [1]. Moreover, whether diabetes is a co–morbidity or a risk factor of cancer is uncertain. In our study only gender and age were controlled when the SIR and SMR were calculated. It was because the information of the life style and anthropometry of the general population in the SCRSS database was absent. The lack of the confounding risk factors was the major shortcoming in our study. Thirdly, the effects of antidiabetic, antineoplastic and other agents on the risks of cancer incidence and mortality were not analyzed. Fourthly, after the T2DM patients enrolled in our center, they would have more healthcare–seeking behaviors during their follow–up time. Besides glucose titrating, the diabetic chronic complications but not cancer screening were their routing examinations. However, they still had more chances to be identified with cancer compared to the general population. Therefore, it may be the predominant reason for the higher risk of cancer in T2DM than general expectation. Finally, the cancer TNM (T, tumor; N, lymph node; M, metastasis) stages in our study were not analyzed which may be associated with cancer mortality. Thus, caution should be taken with the interpretation of our results, and a more detailed, larger and longer multicenter study is needed in future study.

## 5. Conclusions

In summary, the results from the present study demonstrated that, compared with the general population in China, T2DM was significantly associated with higher cancer incidence and mortality risk, especially pancreatic cancer. Additional screening for early stage cancer will be valuable in T2DM patients.

## Figures and Tables

**Table 1:** Comparison of characteristics at baseline between male and female patients.

Characteristics	Male *n* = 6488	Female *n* = 5788	*p*
Age, year	60.2 (18.0)	61.9 (17.0)	<0.0001
Duration of diabetes, years	4.6 (9.3)	6.3 (9.5)	<0.0001
Smoking exposure, no. (%)	2615 (40.3)	113 (2.0)	<0.0001
Diabetic family history, no. (%)	2399 (37.0)	2270 (39.2)	0.008
SBP, mmHg	130.0 (25.0)	140.0 (30.0)	<0.0001
BMI, kg/m^2^	24.2 (4.0)	24.1 (4.7)	0.332
HbA1c, %	7.7 (3.1)	7.4 (2.5)	<0.0001
Microvascular complication, no. (%)	1218 (18.8)	1107 (19.1)	0.619
Macrovascular complication, no. (%)	821 (12.7)	770 (13.3)	0.333

Data are medians (inter-quartile range) for non-normal distributed variables and no. (%) for categorical variables. Wilcoxon signed ranks test for non-normal distributed variables, χ^2^ test for proportions. SBP: systolic blood pressure; BMI: body mass index.

**Table 2:** Follow-up time of the T2DM patients.

Characteristics		Patients Number (%)	Person Years	Median Years of Follow-Up Time (Mean)
Gender	Male	6488 (52.9)	29,294.3	4.2 (4.7)
	Female	5788 (47.1)	26,261.1	4.2 (4.6)
Age at baseline, years (Mean ± SD: 60.7 ± 12.4)	<45	1105 (9.0)	5163.8	4.3 (4.4)
	45–54	2661 (21.7)	12,283.7	4.3 (4.6)
	55–64	3657 (29.8)	16,062.3	4.0 (4.4)
	65–74	3178 (25.9)	14,652.9	4.4 (3.7)
	≥75	1675 (13.6)	7292.8	4.0 (3.8)
Period of enrollment	2001–2005	5207 (42.4)	23,651.9	4.3 (4.5)
	2006–2010	7069 (57.6)	31,803.5	4.1 (4.5)
Diabetic duration at baseline, years (Median, IQR: 5.4, 9.4)	<1.0	2873 (23.4)	13,033.6	4.2 (4.5)
	1.0–4.9	2906 (23.7)	13,189.0	4.2 (4.5)
	5.0–9.9	2833 (23.1)	12,841.7	4.2 (4.5)
	≥10.0	3664 (29.8)	16,391.2	4.2 (4.5)

**Table 3:** Incidence rate of cancer and mortality among diabetic patients.

Outcomes	Total		Male		Female		χ^2^ Test	Cox Analyze (Male *vs.* Female)	
	Number of Cases	Incidence Rate/10^5^ Person-Year	Number of Case	Incidence Rate/10^5^ Person-Year	Number of Case	Incidence Rate/10^5^ Person-Year	*p*	HR (95% CI)	Adjusted HR * (95% CI)
**Cancer**									
Overall cancer	404	728.5	226	774.1	178	677.8	0.206	0.87 (0.72–1.06)	1.00 (0.78–1.28)
Lung cancer	63	113.6	31	106.2	32	121.9	0.574	1.14 (0.70–1.87)	1.26 (0.66–2.39)
Colorectal Cancer	50	90.2	34	116.5	16	60.9	0.031	0.52 (0.29–0.94)	0.63 (0.31–1.24)
Gastric cancer	50	90.2	32	109.6	18	68.5	0.112	0.62 (0.35–1.10)	0.63 (0.32–1.25)
Liver cancer	37	66.7	28	95.9	9	34.3	0.005	0.36 (0.17–0.75)	0.57 (0.24–1.37)
Pancreatic cancer	31	55.9	15	51.4	16	60.9	0.627	1.18 (0.58–2.39)	1.66 (0.66–4.19)
Prostate cancer (men)	-	-	19	65.1	-	-	-	-	-
Breast cancer (women)	-	-	-	-	28	106.6	-	-	-
**Mortality**									
All-cause mortality	779	1402.2	411	1426.9	368	1399.4	0.958	1.00 (0.86–1.15)	1.04 (0.85–1.27)
Death from cancer	196	352.8	113	386.2	83	315.6	0.175	0.82 (0.61–1.08)	0.95 (0.66–1.36)
Lung cancer	47	84.6	24	82.0	23	87.5	0.427	1.06 (0.60–1.88)	0.89 (0.44–1.79)
Colorectal Cancer	16	28.8	11	37.6	5	19.0	0.566	0.46 (0.16–1.31)	0.43 (0.13–1.47)
Gastric cancer	23	41.4	15	51.3	8	30.4	0.591	0.59 (0.25–1.39)	0.62 (0.23–1.71)
Liver cancer	26	46.8	20	68.4	6	22.8	0.792	0.33 (0.13–0.83)	0.62 (0.21–1.83)
Pancreatic cancer	27	48.6	12	41.0	15	57.0	0.252	1.38 (0.65–2.96)	1.58 (0.61–4.07)
Prostate cancer (men)	-	-	4	13.7	-	-	-	-	-
Breast cancer (women)	-	-	-	-	2	7.6	-	-	-

* Adjusted for age, smoking, diabetic duration, BMI, HbA1c, and SBP.

**Table 4:** Standardized cancer incidence ratios in patients with type 2 diabetes.

Cancer Type	Male			Female		
	Obs	Exp	SIR (95% CI)	Obs	Exp	SIR (95% CI)
Overall cancer	226	71.90	3.14 (2.73–3.56)	178	41.49	4.29 (3.64–4.94)
Lung cancer	31	17.84	1.74 (1.13–2.34)	32	5.60	5.71 (3.74–7.69)
Colorectal Cancer	34	8.66	3.93 (2.61–5.24)	16	6.25	2.56 (1.30–3.81)
Gastric cancer	32	10.21	3.13 (2.06–4.21)	18	4.30	4.19 (2.25–6.13)
Liver cancer	28	7.26	3.86 (2.43–5.29)	9	2.53	3.56 (1.15–5.97)
Pancreatic cancer	15	2.75	5.46 (2.69–8.22)	16	1.78	9.00 (4.59–13.41)
Prostate cancer	19	3.47	5.38 (3.01–7.05)	-	-	-
Breast cancer	-	-	-	28	6.08	4.60 (2.90–6.31)

Exp: expected case number; Obs: observed case number; SIR: standardized incidence ratio. Expected cancer cases were based on estimates of the general population in Shanghai, after adjustments for age and gender.

**Table 5:** Standardized mortality ratios in patients with type 2 diabetes.

Cause of Mortality	Male			Female		
	Obs	Exp	SMR (95% CI)	Obs	Exp	SMR (95% CI)
All-cause mortality	411	218.17	1.88 (1.70–2.07)	368	225.87	3.57 (2.78–4.35)
Cancer mortality	113	49.82	2.27 (1.86–2.68)	83	23.26	1.86 (1.46–2.26)
Lung cancer	24	15.79	1.52 (0.91–2.13)	23	4.70	4.90 (2.90–6.90)
Colorectal Cancer	11	4.76	2.31 (0.95–3.67)	5	2.82	1.77 (0.22–3.32)
Gastric cancer	15	7.00	2.14 (1.06–3.22)	8	2.72	2.95 (0.91–4.99)
Liver cancer	20	6.10	3.28 (1.85–4.71)	6	2.12	2.83 (0.57–5.10)
Pancreatic cancer	12	2.76	4.35 (1.88–6.82)	15	1.93	7.76 (3.83–11.69)
Prostate cancer	4	1.38	2.90 (0.06–5.74)	-	-	-
Breast cancer	-	-	-	2	1.53	1.31 (0.00–3.12)

Exp: expected case number; Obs: observed case number; SMR: standardized mortality ratio. Expected cancer cases were based on estimates of the general population in Shanghai, after adjustments for age and gender.
